# Identification of genomic characteristics and selective signals in Guizhou black goat

**DOI:** 10.1186/s12864-023-09954-6

**Published:** 2024-02-09

**Authors:** Lingle Chang, Yundi Zheng, Sheng Li, Xi Niu, Shihui Huang, Qingmeng Long, Xueqin Ran, Jiafu Wang

**Affiliations:** 1 Institute of Agro-Bioengineering/Key Laboratory of Plant Resource Conservative and Germplasm Innovation in Mountainous Region and Key Laboratory of Animal Genetics, Breeding and Reproduction in the Plateau Mountainous Region (Ministry of Education), College of Life Sciences and College of Animal Science, Guizhou University, Guiyang, 550025 Guizhou China; 2 Guizhou Testing Center for Livestock and Poultry Germplasm, Guiyang, 550018 Guizhou China

**Keywords:** Guizhou black goat, Whole-genome sequencing, Genetic diversity, Selection signature

## Abstract

**Background:**

Guizhou black goat is one of the indigenous black goat breeds in the southwest region of Guizhou, China, which is an ordinary goat for mutton production. They are characterized by moderate body size, black coat, favorite meat quality with tender meat and lower odor, and tolerance for cold and crude feed. However, little is known about the genetic characteristics or variations underlying their important economic traits.

**Results:**

Here, we resequenced the whole genome of Guizhou black goat from 30 unrelated individuals breeding in the five core farms. A total of 9,835,610 SNPs were detected, and 2,178,818 SNPs were identified specifically in this breed. The population structure analysis revealed that Guizhou black goat shared a common ancestry with Shaanbei white cashmere goat (0.146), Yunshang black goat (0.103), Iran indigenous goat (0.054), and Moroccan goat (0.002). However, Guizhou black goat showed relatively higher genetic diversity and a lower level of linkage disequilibrium than the other seven goat breeds by the analysis of the nucleotide diversity, linkage disequilibrium decay, and runs of homozygosity. Based on *F*_ST_ and θ_π_ values, we identified 645, 813, and 804 selected regions between Guizhou black goat and Yunshang black goat, Iran indigenous goat, and cashmere goats. Combined with the results of XP-EHH, there were 286, 322, and 359 candidate genes, respectively. Functional annotation analysis revealed that these genes are potentially responsible for the immune response (e.g., *CD28*, *CD274*, *IL1A*, *TLR2*, and *SLC25A31*), humility-cold resistance (e.g., *HBEGF*, *SOSTDC1*, *ARNT*, *COL4A1/2*, and *EP300*), meat quality traits (e.g., *CHUK*, *GAB2*, *PLAAT3*, and *EP300*), growth (e.g., *GAB2*, *DPYD*, and *CSF1*), fertility (e.g., *METTL15* and *MEI1*), and visual function (e.g., *PANK2* and *NMNAT2*) in Guizhou black goat.

**Conclusion:**

Our results indicated that Guizhou black goat had a high level of genomic diversity and a low level of linkage disequilibrium in the whole genome. Selection signatures were detected in the genomic regions that were mainly related to growth and development, meat quality, reproduction, disease resistance, and humidity-cold resistance in Guizhou black goat. These results would provide a basis for further resource protection and breeding improvement of this very local breed.

**Supplementary Information:**

The online version contains supplementary material available at 10.1186/s12864-023-09954-6.

## Background

The goat (*Capra hircus*) is a domesticated species of goat-antelope typically kept as livestock. It is one of the oldest domesticated species of animal, according to archaeological evidence that its earliest domestication occurred 10,000 calibrated calendar years ago in Iran [1]. With the domestication of goats, both natural and artificial selection led to the formation of breeds with distinct phenotypic characteristics including morphological, physiological, and adaptive traits. There are 557 domesticated goat breeds distributed all over the world [2, 3], and 58 indigenous breeds adapted to different agroclimatic conditions in China [4]. Selection signatures, as selective imprints such as genetic polymorphism reduction and linkage disequilibrium left on the genome by natural and/or artificial selection, can affect the agronomic and adaptive traits of domesticated animals and have been well detected by whole genome sequencing (WGS) [5–7]. WGS gradually turned to the study of indigenous breeds with obvious adaptive traits, such as Chinese goat breeds [3, 8–11]. These goat breeds were identified with abundant genomic imprints adapting to local environments or livestock demand, such as disease resistance, altitude adaptation, coat color, growth, milk quality, and reproductive performance [3, 8–10, 12]. Chen et al. identified selective signatures related to anti-skin diseases in Longlin goats through WGS [13]. The above evidence can provide choices for developing genetic breeding programs to improve the productivity and adaptability of Chinese indigenous goat breeds.

As an indigenous goat breed, which is marked as a special agricultural product with geographical indications registered by the State Administration for Industry and Commerce of China, GZB is mainly used for mutton production in the karst mountainous region of the Yunnan-Kweichow plateau. Because of the natural geographical isolation formed by the special karst terrain, the gene exchange process between GZB and other goat breeds was blocked for a long period, and this breed has been propagated and preserved up to now. Compared with artificial selection, GZB bears more pressure of natural selection, which makes them have a strong living ability, and a unique set of body structures and physiological mechanisms for roughness, humidity, and cold resistance. Meanwhile, this indigenous goat breed might carry alleles that enable them to adapt to local conditions. But the genomic characteristics or variations underlying its adaptive and agronomic traits have still not been investigated.

In the present study, we aimed to explore the genomic characteristics and selection signatures in the genome of GZB. Utilizing WGS and genomic approaches, we conducted the first comparative genomic study to reveal the genome-wide variation and selection signatures in the GZB population.

## Results

### Sequencing and identification of SNPs

**Fig. 1:**
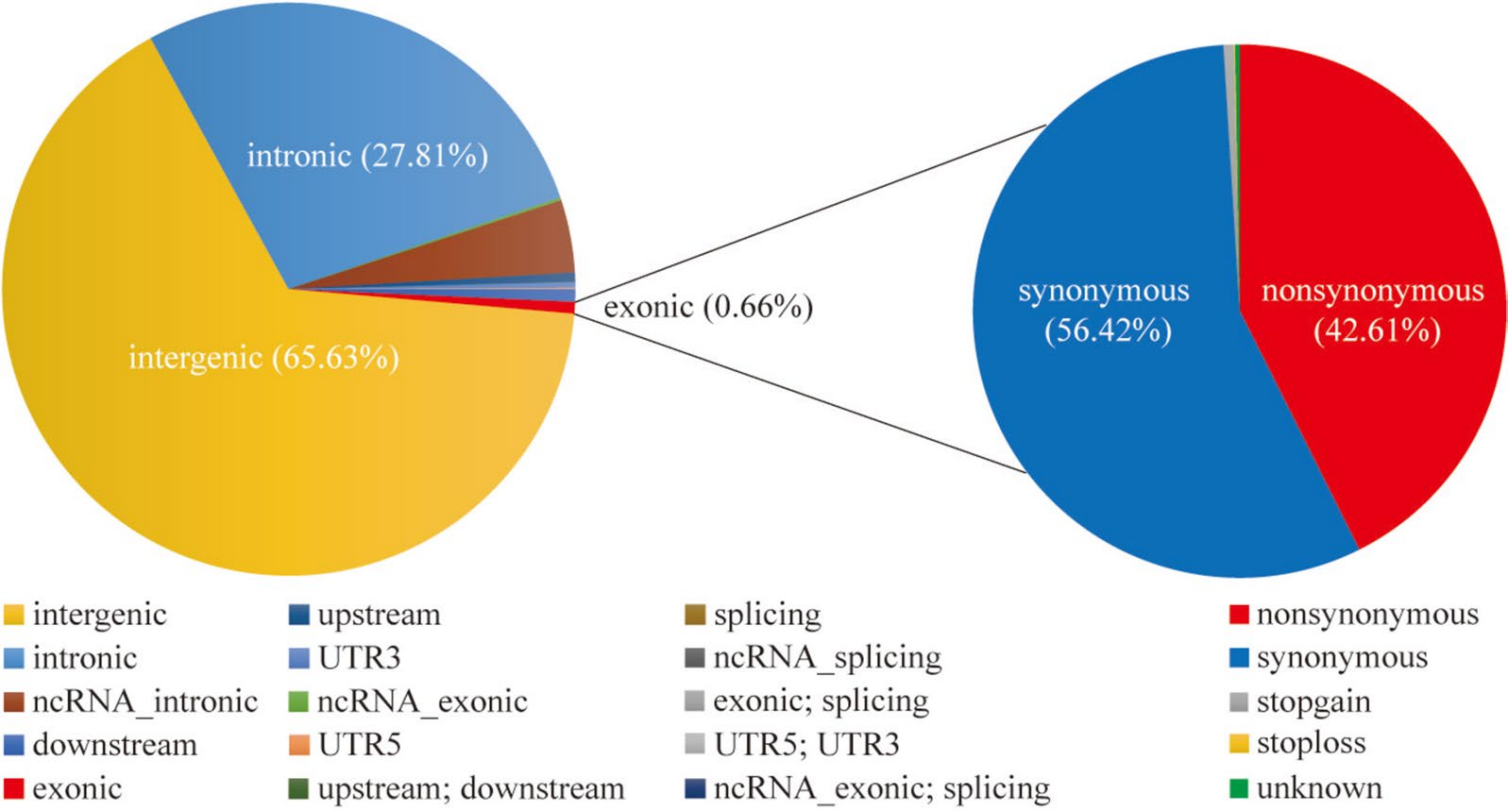
Statistics regarding the whole genome SNP variant types of GZB using ANNOVAR. Plot of the total variant annotation and coding consequence variant annotation

High-throughput sequencing generated genomic data for 30 GZB (Supplementary Table S1) at an average sequencing depth of approximately 15.3-fold. It was jointly genotyped with 79 (Supplementary Fig. 1, Supplementary Table S2) publicly available genomic data from eight representative populations, including Iran indigenous goat (IIG, *n* = 15), South Korean goat (SKG, *n* = 12), Moroccan goat (MG, *n* = 12), Yunshang black goat (YBG, *n* = 11), French goat (FG, *n* = 10), Shaanbei white cashmere goat (SCG, *n* = 9), Wild goat (WG, n = 6), and Tibetan goat (TG, n = 4). The total number of SNPs detected within the populations was shown in Supplementary Table S3. Then we annotated 9,835,610 biallelic SNPs that were discovered in 30 GZB, and GZB had the highest number of SNPs among nine populations (Supplementary Table S3). Functional annotation of the polymorphic sites revealed that the vast majority of SNPs were present in either intergenic regions (65.63%) or intronic regions (27.81%). Exons contained 0.66% of the total SNPs with 46,767 (42.61%) nonsynonymous SNPs and 61,917 (56.41%) synonymous SNPs (Fig. 1, Supplementary Table S4). We also found that the shared SNP counts of nine goat populations were 2,783,053 (Supplementary Fig. 2a), while the unique SNP counts of GZB weres 2,178,818 (Supplementary Fig. 2b).

### Population structure of Guizhou black goat and other eight goat populations

**Fig. 2:**
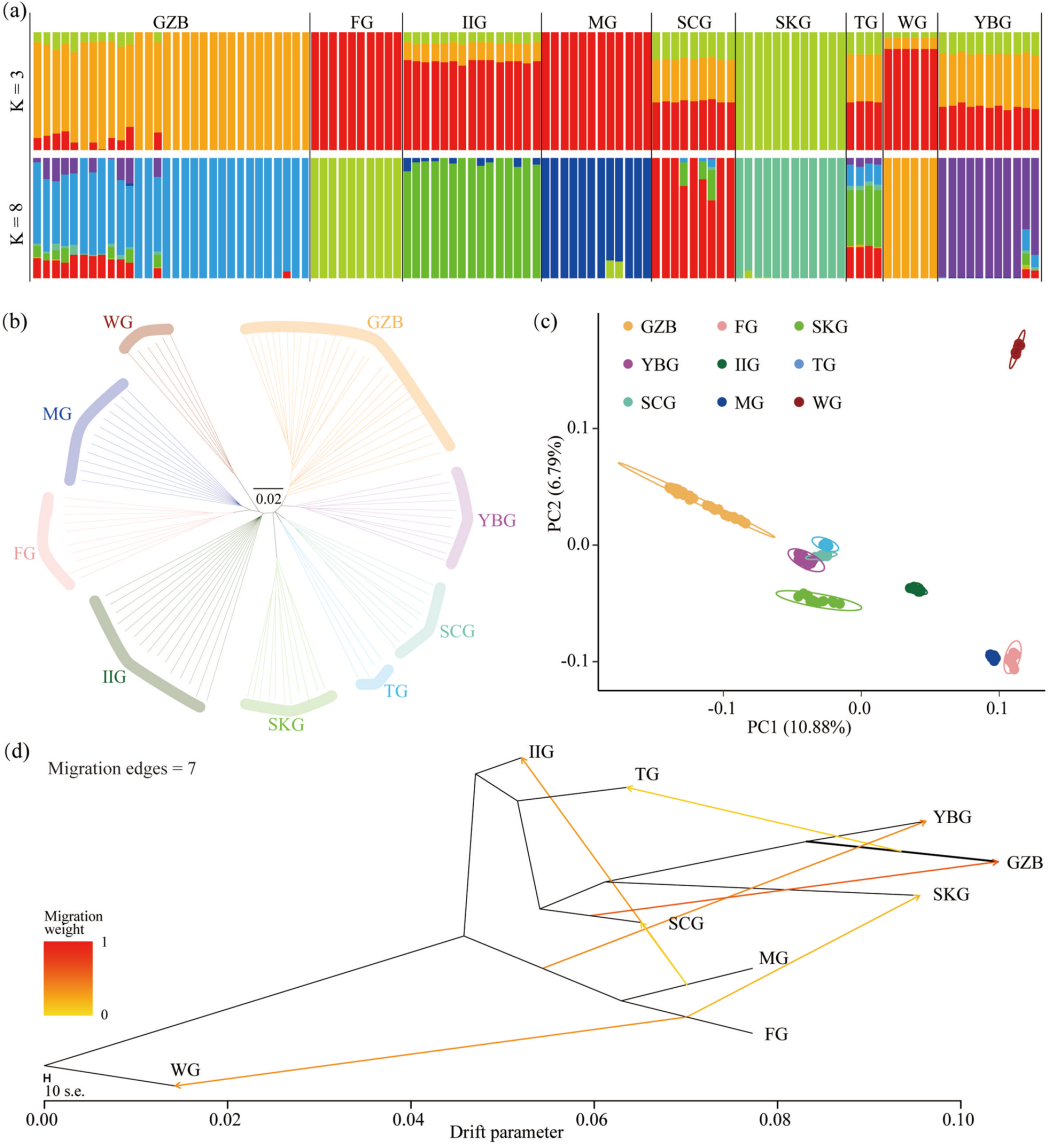
Population Structure of Guizhou Black Goat and its relationship with other eight goat populations in the world. **a** Model-based clustering of goat populations using ADMIXTURE. The length of each colored segment represents the proportion of the individual’s genome from K = 3 and K = 8 ancestral populations. The population names are at the top of the figure. **b** Neighbor-joining phylogenetic tree of the nine goat populations. The scale bar represents proportional to similarity (*p* distance). **c** Principal component analysis of nine goat populations. Different colored lines or points represent different categories. GZB (Guizhou black goat), YBG (Yunshang black goat), SCG (Shaanbei white cashmere goat), TG (Tibetan goat), SKG (South Korean goat), IIG (Iran indigenous goat), WG (Wild goat, *Capra aegagrus*), MG (Moroccan goat), and FG (French goat). **d** ML tree of nine goat populations with migration edges = 7

To explore relatedness among GZB and other goat populations distributed worldwide, we conducted ADMIXTURE, neighbor-joining (NJ) tree, principal component analysis (PCA), and Maximum likelihood (ML) tree using whole-genome SNP data (Fig. 2). The ADMIXTURE analysis revealed K = 3 (cross-validation error = 0.52634) as the most likely number of genetically distinct populations for nine goat populations (Supplementary Table S5). When K = 8, some GZB showed clear evidence of genetic heterogeneity with shared genome ancestry with SCG (0.146), YBG (0.103), IIG (0.054), and MG (0.002) genetic background (Fig. 2a, Supplementary Table S6). The NJ tree recapitulated these findings and showed that the genetic distance between GZB and other goat populations became farther with the geographical distance. GZB was found to be genetically closer to YBG (Fig. 2b). The PCA (Fig. 2c) showed similar results with NJ tree, which together revealed genetic differences between GZB and other goat populations at the overall genomic level. ML tree analysis showed that when the number of migration events was seven, GZB had gene flow from SCG, and flowed out to TG (Fig. 2d).

### Genetic diversity, and linkage disequilibrium of nine goat populations

**Fig. 3:**
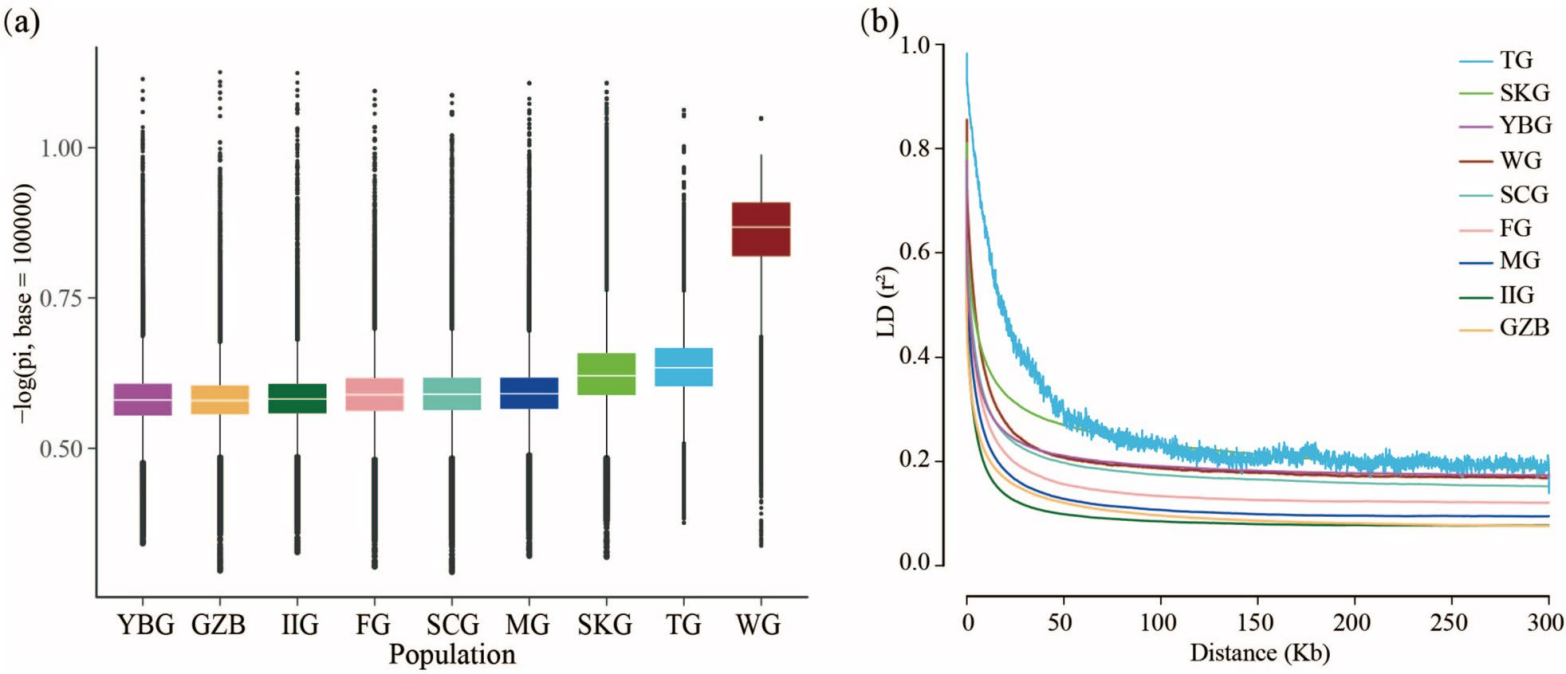
**a** Genome-wide distribution of nucleotide diversity of each population in 100 kb windows with 10 kb steps. The horizontal line inside the box indicates the median of this distribution; box limits indicate the first and the third quartiles, and points show outliers. Data points outside the whiskers can be considered outliers. **b** Genome-wide average LD decay estimated from each population

To examine the degree of nucleotide sequence variation among individuals in each goat population, nucleotide diversity was calculated. The results showed that nucleotide diversity was the highest in YBG (0.001376), tightly followed by GZB (0.001352) (Fig. 3a). In contrast, we observed a lower value of LD in GZB, following IIG closely (Fig. 3b).

### Detection of selection signals and selective sweep in GZB

Based on the results of population genetic structure, distinct agronomic traits, and environmental characteristics of production areas, the goat populations were regrouped into three representative populations: Yunshang black goat (muttony goat breed), Iran indigenous goat (living in arid or semi-arid areas), and Cashmere goat (CG, Shaanbei white cashmere goat and Tibetan goat for cashmere production).

**Fig. 4:**
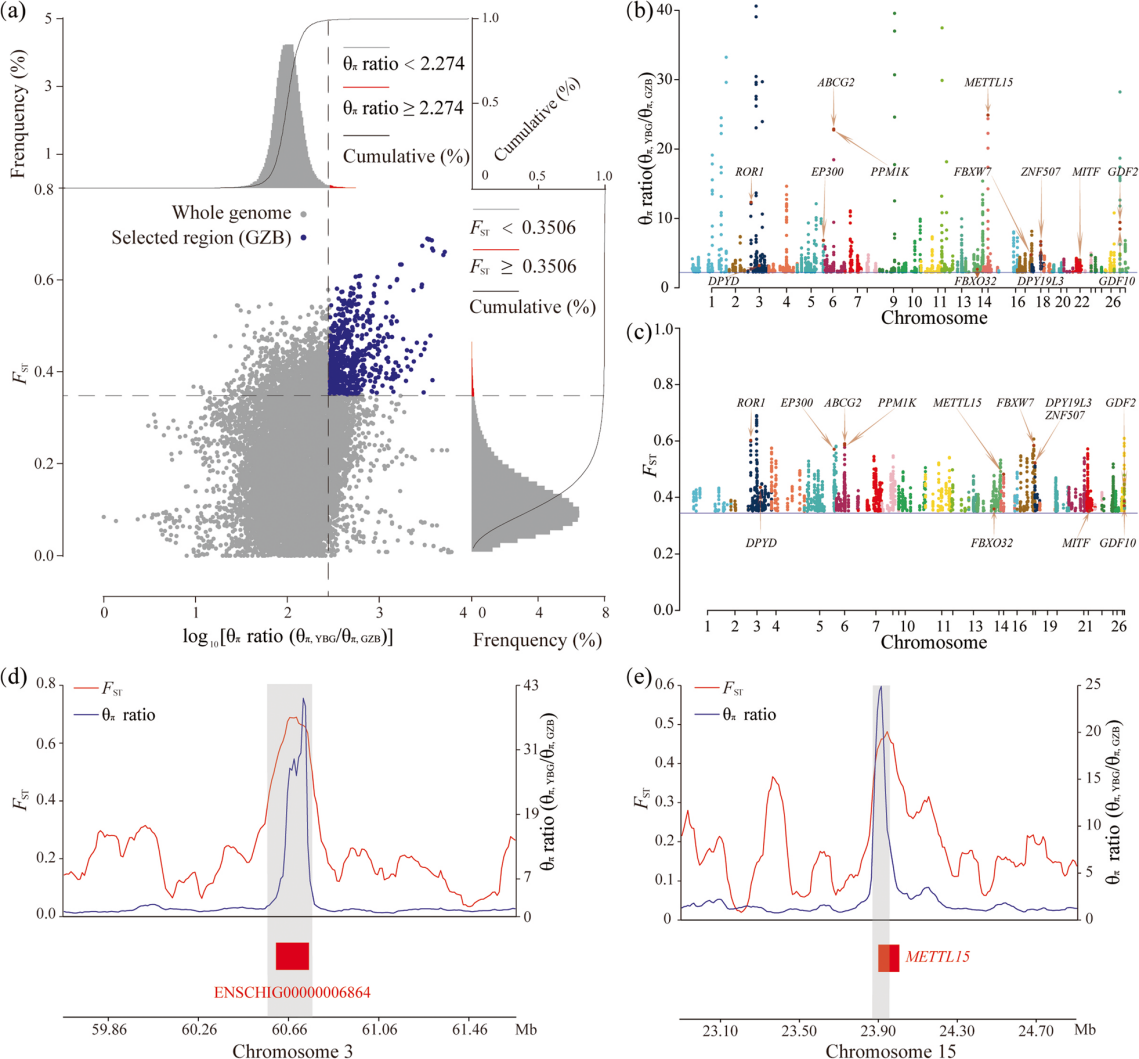
Analysis of the signatures of positive selection in the genome of GZB compared to YBG. **a** Distribution of θ_π_ ratios (θ_π, YBG_/θ_π, GZB_) and *F*_ST_ values, which are calculated in 100 kb windows sliding in 10 kb steps. Data points located to the right of the vertical dashed lines, respectively (corresponding to the 1% right tail of the empirical θ_π_ ratio distribution, where the θ_π_ ratio is 2.274), and above the horizontal dashed line (the 1% right tail of the empirical *F*_ST_ distribution, where *F*_ST_ is 0.3506) were identified as selected regions for GZB (blue points). **b** Manhattan plot of selective sweeps using θ_π_ ratio in GZB vs. YBG. Solid blue line represented the threshold of the top 1% θ_π_ ratios. **c** Manhattan plot of selective sweeps using *F*_ST_ in GZB vs. YBG. Solid blue line represented the threshold of the top 1% *F*_ST_ values. **d-e** Example of genes with strong selective sweep signals in GZB. θ_π_ ratio and *F*_ST_ values are plotted using a 10 kb sliding window. Gray rectangle regions were termed as regions with strong selective sweep signals for GZB. The boundaries of ENSCHIG00000006864 and *METTL15* genes are marked in red

**Fig. 5:**
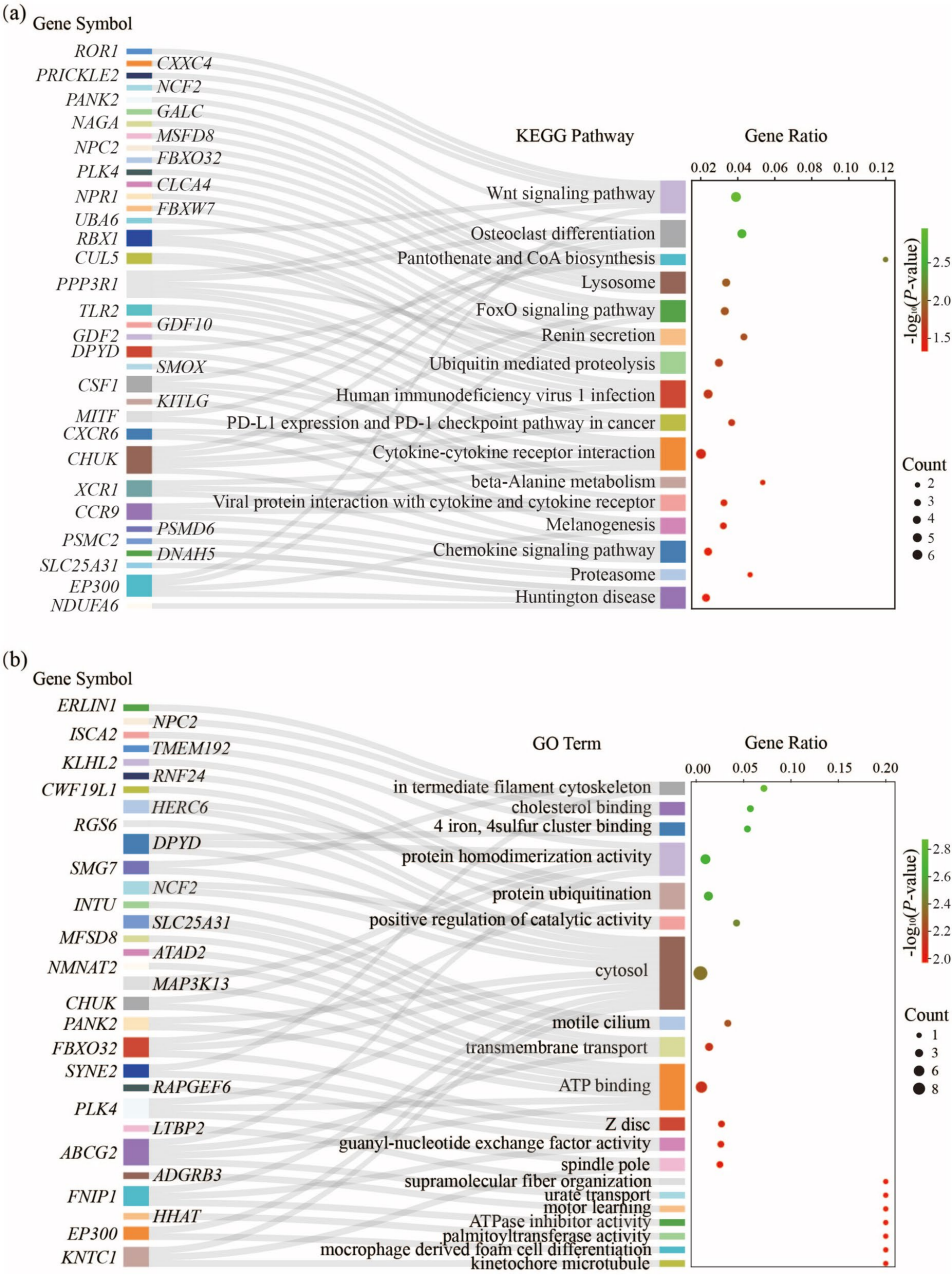
GO and KEGG pathway enrichment analysis shows significant (*P* < 0.05) terms, pathways, and associated genes in GZB vs. YBG comparison. **a** The Sankey-Dot plot of the 16 significant KEGG pathways. **b** The Sankey-Dot plot of the Top 20 GO terms. The size of circles for each pathway represents counts of associated genes. The color of the circles indicates the *P*-value

Combined *F*_ST_, θ_π_, and XP-EHH, we detect genomic regions associated with selection in the GZB with YBG, IIG, and CG, respectively. And we selected the top 1% of signals as candidate regions. The 645 selected regions (blue points) were detected and 252 candidate genes were extracted in the GZB vs. YBG comparison (Fig. 4a-c, Supplementary Fig. 3, and Supplementary Table S7). By adding the XP-EHH to detect among-population selection signals, we obtained 34 selected genes different from the results of the above two methods (Supplementary Fig. 4a). And 258 GO terms and 22 KEGG pathways were significantly enriched (*P* < 0.05, Fig. 5a-b, Supplementary Table S8-S9). Of which, growth/development-related terms have a high rate of occurrence. The Wnt signaling pathway (*P* = 0.001) is indispensable in the growth and development, involving *PRICKLE2*, *PPP3R1*, *CXXC4*, *RBX1*, *EP300*, and *ROR1*. Ten GO terms (e.g., osteoblast differentiation, growth factor activity, and limb development) are also significantly enriched in growth/development (*P* < 0.05). In addition, a region of 0.29 Mb on chromosome 3 containing ENSCHIG00000006864 (novel gene, RNA gene, lncRNA) was strongly selected by *F*_ST_ (average *F*_ST_ = 0.604) and θ_π_ ratio (average θ_π_ ratio = 17.1) (Fig. 4d). Moreover, we noticed *METTL15*, which is related to mitochondrial rRNA methylation (rRNA base methylation and mitochondrial matrix), showing a strong positive selection signal in GZB (Fig. 4e). A missense mutation (rs648661574, c.A60C, p.E20D) was found at *METTL15* gene. This mutation presented a huge divergence between GZB (allele C frequency = 0.9) and YBG (allele A frequency = 1).

In the selection signals and selective sweep analysis between GZB and IIG, 813 selected regions and 324 candidate genes were scanned by *F*_ST_ and θ_π_ (Supplementary Fig. 3, Supplementary Fig. 5a-c, and Supplementary Table S7). We obtained 32 selected genes by adding the XP-EHH (Supplementary Fig. 4b). In KEGG pathway enrichment analysis, the top 1 was osteoclast differentiation (*P* = 0.001), and 17 (17/32) immune-related pathways (e.g., rheumatoid arthritis, Human papillomavirus infection, and type I diabetes mellitus) were enriched (*P* < 0.05, Supplementary Table S10). The selected gene *IL1A* (Supplementary Fig. 5d) is involved in six of these pathways and is associated with disease. In GO enrichment analysis, we got 281 significant terms (*P* < 0.05, Supplementary Table S11). There were several significant terms related to environmental adaptation, such as cellular response to UV and cellular response to heat. In addition, a region of 0.24 Mb on chromosome 4 (containing *DNAJC2*, *PMPCB*, *PSMC2*, *RELN*, and *SLC26A5*) was strongly selected by *F*_ST_ and θ_π_ ratio (Supplementary Fig. 5e), and a nonsynonymous SNV (novel variant, c.A4G, p.T2A) was found in the *PMPCB* gene. Allele G displayed an abundant distribution (frequency = 1) in GZB, whereas it showed an opposite pattern (frequency = 0) in IIG.

A total of 804 selected regions and 359 candidate genes were found in the comparison of GZB and CG (Supplementary Fig. 3, Supplementary Fig. 4c, Supplementary Fig. 6a-c, and Supplementary Table S7), and eleven of them were significantly enriched six fiber-related terms (e.g., keratin filament, cornification, elastic fiber, stress fiber, and regulation of keratinocyte proliferation) (*P* < 0.05, Supplementary Table S12). Moreover, two missense mutations (rs667703315, c.G1511A, p.S504N. rs649013003, c.A2680G, p.N894D) were found at the *KRT79* and *PRKD1* genes among eleven genes, respectively (Supplementary Fig. 6d-e). As one of the peculiar selected genes in this comparison group, *JAK2*, containing a nonsynonymous SNV (rs647159917, c.G1573A, p.A525T) is involved in multiple immune-related pathways (e.g., influenza A, Th1 and Th2 cell differentiation, and Th17 cell differentiation) (*P* < 0.05, Supplementary Table S13).

### Variant accuracy

We inspected 11 selected SNPs (Supplementary Table S14) in candidate functional genes below from 30 individuals obtained by the Sanger sequencing approach, giving an overall validation rate of 99.39%. Overall, the results indicated confidence in the correct rate of variant calling of SNP.

## Discussion

Characterizing population structure and genetic diversity is essential for the revelation of evolutionary history, understanding of environmental adaptation, conservation and utilization of germplasm resources, and investigation of phylogenetic relationships. In this study, we performed a whole-genome resequencing analysis of 30 GZB. This is the hitherto most comprehensive data set on the population genetic structure of GZB. Neither the number of individuals nor the depth of sequencing used in previous studies is comparable to this study. We found that GZB had the highest number of SNPs among the nine populations. This may be related to the number of samples and the depth of whole genome resequencing. Then we explored the population genetic structure of GZB in the context of the goat populations with potential ancestors and identified useful nonsynonymous SNPs that involved local adaptation and agronomic traits. As shown in Fig. 2a, nearly half (13) of the GZB contained ancestral contributions from SCG (~ 14.6%), YBG (~ 10.3%), and IIG (~ 5%), and more than half (17) of the GZB with pure genetic background seemed to have originated locally in Guizhou, China. In other words, we needed to integrate more in-depth information to confirm the origin of GZB. Meanwhile, the habitation and relationship of GZB was nearby to the YBG in southwest China (Fig. 2b-c). GZB and YBG (mean θ_π_ = 0.001376) showed a similar level of nucleotide diversity, which may be related to their similar genetic background (Fig. 3a). The relatively high level of genomic diversity found in GZB might reflect the stronger selection pressure and longer selection history. In addition, the patterns of LD decay in each population were largely consistent with the results of nucleotide diversity. The above results confirmed that the GZB harbored fewer variants, lower linkage decay, and higher nucleotide diversity comparable to the other native populations, suggesting unique genetic characteristics.

The typical characteristic of GZB is moderate in body size, approximately 55 cm in height, and ~ 30 kg in weight at one year old [14]. Body size is a key factor in determining mutton production. When analyzing the selection signatures of GZB with the bigger YBG (weighing 46 kg at one year old), several positively selected genes were detected associate with growth (*SUOX*, *CSF1*, *CHUK*, *DPYD*, and *GDF2*) and fatty acid metabolism (*GAB2*, *SMOX*, and *GOT2*). Sulfite oxidase (SUOX) plays an important role in bovine bone development [15]. *CSF1* is involved in the fast growth rate of large white pigs at an early stage [16] and is an essential growth factor for osteoclast progenitors and an important regulator for bone resorption [17]. A previous study suggests that *CHUK* has an intrinsic cell-autonomous role in chondrocytes that controls chondrocyte phenotype and affects ontogeny [18]. *DPYD* is related not only to muscle growth but also to fat deposition [19]. Previous studies have demonstrated that GDF2 is the most potent bone morphogenetic protein that can be used to induce bone formation from mesenchymal stem cells both in vitro and in vivo, through a comprehensive analysis of osteogenic activity [20–22]. *Gab2* plays an important role in regulating adipocyte maturation, differentiation, and function by using mouse primary or immortalized brown preadipocytes in vitro [23]. *SHOX* is considered to be involved in the physiological processes of sheep growth and carcass composition traits [24]. GOT2 can affect pork quality by participating in aromatic amino acid metabolism [25]. *PLAAT3* has been found to be involved in regulation of lipogenesis in mice, pigs and humans, and is associated with neurological manifestations in human cases [26–28]. These selected genes described above might be involved in regulating the growth and forming special mutton quality traits of GZB.

The population expansion of GZB greatly depends on high fecundity. Although crossbreeding performance has improved fecundity in the recent two decades, long-term crossbreeding is not conducive to the protection of germplasm resources. Therefore, we should look for genes and loci related to lambing traits, to guide the purification and rejuvenation of GZB by molecular biological technology. The fecundity of YBG selected by hybridization has been greatly improved compared with the female parent (Yunling black goat). In comparison between GZB and YBG, some candidate genes (*MEI1*, *PANK2*, and *CCDC63*) were significantly enriched in reproduction-related terms (“meiosis I, GO:0007127” and “spermatid development, GO:0007286). Of which, *MEI1* is involved in the regulation of meiosis, and its variation can lead to oocyte developmental disorders and azoospermia [29–31]. In addition, *METTL15* (rs648661574, c.A60C, p.E20D) gene, involved in mitochondrial rRNA methylation, shows a strong positive selection signal in GZB. Considering that mitochondria are maternally inherited in most mammals [32], and ATP produced by oocyte mitochondria is the most direct energy during spindle formation and oocyte fertilization, we can reasonably infer that *METTL15* may regulate oocyte maturation or embryo development by regulating mitochondrial biogenesis, directly or indirectly affecting fertility of GZB.

High disease resistance is a prominent characteristic of GZB. The GZB is generally more resistant to various diseases than commercial goat breeds. However, there are few reports related to the disease of GZB in the production and scientific research up to now. Our selection signal analysis identified several noteworthy genes involved in 17 immune-related pathways, in particular the *CD274*, *SIGLEC1*, *COL4A1/2*, *CHUK*, *PTPRN2*, and *TLR2*. *CD274* is considered to be a host-targeted therapeutic target for *Mycoplasma bovis* infection in cattle [33]. Upregulation of the *SIGLEC1* feedback loop induced by a viral infection can inhibit type I IFN production and suppress antiviral innate immune responses [34]. Mice completely deficient in *Col4a1* and *Col4a2* (*Col4a1*^-/-^, *Col4a2*^-/-^) died at mid-gestation with various defects, including neuronal ectopia, capillary network disorder, and impaired placental development [35]. *Col4a1* mutant mice shows thinning and fragmentation of the glomerular basement membrane and functional kidney pathology, including microalbuminuria and hematuria [36]. Skin, as the largest organ of animals, is the first line of immune defense. Earlier studies have shown that *CHUK* (Component of Inhibitor of Nuclear Factor Kappa B Kinase Complex) is essential for NF-κB activation in limbs and skin during embryonic development [37]. Loss of *CHUK* interferes with a variety of morphogenetic events, including patterning of limbs and bones and proliferation and differentiation of epidermal keratinocytes [38, 39]. Cell and animal experiments show that HOXD13 can bind to *PTPRN2* (Protein tyrosine phosphatase, receptor type N2) promoter and up-regulate its expression, thus promoting the development of colon cancer [40]. Epigenetic studies confirm lower methylation levels of *PTPRN2* in African-American lupus patients compared with those of European-American descent, which suggests its role for immune response [41]. *PTPRN2* encodes a major islet autoantigen in type 1 diabetes and is involved in the regulation of obesity mechanisms. CpG methylations of *PTPRN2* gene is closely associated with childhood obesity under certain genetic background. This suggests that genetic and epigenetic interactions of *PTPRN2* gene may be involved in regulating childhood obesity [42]. *TLR2* (Toll Like Receptor 2) participates in oxidative damage in dairy goats [43], anti-inflammation in bovine mammary epithelial cells [44], heat stress immunomodulation in black Bengal goats [45], and rheumatoid arthritis in humans [46]. In addition, we observed two nonsynonymous SNPs (rs666613706, c.T65C, p.V22A; rs650082970, c.A757G, p.I253V) on *TLR2* with a high frequency in GZB. Theoretically, we might assume that these genes, nonsynonymous SNPs, and pathways was subject to selection for immune-related traits such as resistance to bacteria and viruses infections in the GZB population.

Given the natural conditions of high humidity (annual mean humidity 75%-80%) and cold (annual average temperature 10 °C) [47] in the plateau mountainous area, GZB may have acquired desirable humidity and cold resistance characteristics through long-term natural selection. We found cold-resistant candidate genes in the GZB, such as *HBEGF*, *SOSTDC1*, *EP300*, *ZNF518B*, and *ARNT*. *HBEGF* may contribute to skin wound healing by participating in autophagy-related pathways [48, 49]. It is suggested that SOSTDC1 secreted from skin lymphatic vessels may act as a paracrine factor of hair follicle growth [50]. And *EP300 *[51] might involve in lipid metabolism in pigs. Silencing Zfp518b (rat homologue of *ZNF518B*) in clonal rat β-cells alters insulin secretion [52]. Blood flow parameters investigated in the mice ARNT*-*knockout mutation indicate the impaired blood flow recovery [53]. These genes can protect against cold and humidity by regulating physiological processes such as wound healing, lipid metabolism, hair follicle dissipation, glycogen metabolism, and blood flow to keep heat balance.

## Conclusion

This study provided the first comprehensive overview of single nucleotide variations in GZB by using WGS data. The results showed that GZB had high nucleic acid diversity and genetic diversity, and it was a relatively independent local goat breed with low artificial selection intensity in the special wet and cold mountain environment. It should be protected scientifically and effectively as a valuable germplasm resource. Moreover, we identified plenty of candidate genes and SNPs that might be responsible for immune response, cold/humidity resistance, mutton quality traits, growth, and fertility of GZB. Overall, this study is of great significance for understanding the genetic diversity and adaptability of goat breeds in the karst region of Southwest China and also provides a basis for studying the genomic characteristics of other important local goat breeds in the world.

## Methods

### Population resequencing

We sampled a total of 30 GZB from five core breeding farms which were respectively located in the Weining and Hezhang counties of Guizhou province, China. Genomic DNA was extracted from the ear tissue of each individual. The whole-genome resequencing was performed by a DNBSEQ-T7 sequencer (BGI, Shenzhen, China) according to the manufacturer’s recommendations. In addition, we downloaded the genome data of 79 individuals across the world from the European Bioinformatics Institute website (www.ebi.ac.uk/), including 15 Iran indigenous goats (PRJEB3135), 12 Morocco goats (PRJEB3134), 12 South Korean goats (PRJNA245770), 11 Yunshang black goats (PRJNA611688), 10 French goats (PRJEB37122), 9 Shaanbei white cashmere goats (PRJNA780339), 6 wild goats (PRJEB3136), and 4 Tibetan goats (PRJNA281979).

### Variant calling

Fastp v0.23.4 [54] were used for assessing a per-base sequence quality with default parameters. The command was like ‘fastp -i /data/A_1.fastq.gz -I /data/A_2.fastq.gz -o /data/A_1.QC.gz -O /data/A_2.QC.gz’. The high-quality 150 bp paired-end reads were aligned to the goat reference genome ARS1, using the Burrows-Wheeler aligner v.0.7.8 software [55] with default parameters, such as bwa mem -R ' @RG\tID:SCG\tSM:A' -t 64 /data/ARS1.fa /data/A_1.QC.gz /data/A_2.QC.gz > A.sam. We then converted the mapping reads into bam files and sorted the files using SAMtools v.1.9 [56] by default parameters, such as samtools view -bS A.sam > A.sort.bam. Duplicates were removed by the MarkDuplicates module in GATK v.4.3.0.0 [57] with command ‘gatk –java-options "-Xmx16g -Djava.io.tmpdir = ./tmp" MarkDuplicates -I A.sort,bam -M A.metrics –CREATE_INDEX -O A.sort.MarkDup.bam’. SNPs and Indels were called from the bam files by the GATK HaplotypeCaller module with the GATK best-practice recommendations [57]. The recommended command was like gatk –java-options "-Xmx4g" HaplotypeCaller -R ARS1.fa -I A.sort.MarkDup.bam -O A.g.vcf.gz. Raw GVCFs with the samples called individually were merged using the CombineGVCFs and genotyped by the GenotypeGVCFs. We then extracted and filtered SNPs using the GATK module SelectVariants. The recommended command was like gatk SelectVariants -R ARS1.dna.toplevel.fa -V output.vcf.gz –select-type-to-include SNP -O raw_snps_genotype.vcf. To avoid potential false-positive calls, we implemented "VariantFiltering" of the GATK for the selected SNPs using the best practice parameters "QUAL > 30.0 || QD < 2.0 || FS > 60.0 || MQ < 40.0 || SOR > 3.0 || MQRankSum < -12.5 || ReadPosRankSum < -8.0". We then filtered out nonbiallelic SNPs. After the quality screening, all the identified SNPs were further annotated using ANNOVAR [58] based on the gene annotations of the goat reference genome ARS1. Locations for SNPs in various genic and intergenic regions and synonymous or nonsynonymous SNPs in exonic regions were annotated.

### Phylogenetic and population genetic analyses

We pruned the SNPs in high levels of pair-wise LD using PLINK [59] with the parameter (–indep-pairwise 50 10 0.2) to perform principal component analysis (PCA) and ADMIXTURE analysis. As PCA, the first two eigenvectors were plotted in the ggplot2 package under the R platform. Population structure analysis was carried out using ADMIXTURE v1.3 [60] with kinship (K) set from 2 to 9. Gene flow analysis was performed using Treemix with m = 5 and i = 10. The unrooted NJ tree was constructed by PLINK using the matrix of pairwise genetic distances and visualized with MEGA X [61] and FigTree v1.4.4 (http://tree.bio.ed.ac.uk). Construction and visualization of the ML tree was done by Treemix [62] v1.1.3. The squared correlation (r^2^) between any two loci was calculated to evaluate Linkage disequilibrium (LD) decay using the PopLDdecay v3.41 [63].

### Calculation of θ_π_, *F*_ST_, and XP-EHH

A sliding-window approach (100 kb windows sliding in 10 kb step size) was applied to quantify polymorphism levels (θ_π_, the ratio of nucleotide diversity) and pairwise genetic differentiation (*F*_ST_) between GZB and other goat populations. The programs were used to calculate θ_π_ and *F*_ST_: vcftools –vcf /data/SNP.vcf –keep /data/GZB.txt –window-pi 100,000 –window-pi-step 10,000 –out /data/GZB_IIG, vcftools –vcf /data/SNP.vcf –keep /data/IIG.txt –window-pi 100,000 –window-pi-step 10,000 –out /data/cll/IIG_GZB; vcftools –vcf /data/SNP.vcf –weir-fst-pop /data/GZB.txt –weir-fst-pop /data/IIG.txt –out /data/GZB_IIG –fst-window-size 100,000 –fst-window-step 10,000. XP-EHH was calculated by chromosome with command: selscan [64] –xpehh –vcf GZB.chr k.vcf –vcf-ref YBG.chr k.vcf –map chr k.MT.map.distance –out chr k.GZB_YBG.out.

### Identification of selected regions

To detect regions with significant signatures of selective sweeps, we divided the 4 goat populations (except for Wild goat, Morocco goat, French goat, and South Korean goat) into three reference populations, namely CG (Cashmere goat, 9 Shaanbei white cashmere goat, 4 Tibetan goat), IIG (15 Iran indigenous goat), YBG (11 Yunshang black goat). To uncover selection signatures of GZB, we calculated pairwise *F*_ST_ and θ_π_ in 100 kb sliding windows with a step size of 10 kb across the autosomes between GZB and YBG, IIG, or CG populations, respectively. The windows with high values of θ_π_ ratio and *F*_ST_, representing the top 1% of all windows, were determined as the selected regions.

### Gene functional enrichment analysis

Kyoto Encyclopedia of Genes and Genomes (KEGG) [65–67] pathways and Gene Ontology (GO) terms were analyzed based on the candidate genes via *F*_ST_ and θ_π_ methods using KOBAS-intelligence [68] to investigate the biological enrichment of genes under selective pressure. The GO terms and KEGG pathways were considered to be significantly enriched only when the *P*-value was less than 0.05.

### SNP validation

To check the confidence of SNPs called, we randomly validated 11 SNPs in specific genes from 30 individuals that were genotyped by PCR and Sanger sequencing. The primers used for PCR were designed with DNAMAN v9.0.1.116 (Lynnon Biosoft, USA). The PCR reactions were carried out in 50 μL volume containing 25 μL of 2 × taq PCR Master Mix (TIANGEN Biotech, Beijing, China), 2 μL (10 pmol/mL) for each forward and reverse primer (Supplementary table S14), 2.5 μL DNA templates (30-100 ng/mL), and the remainder supplied with dd H_2_O. The reactions were performed by a BIO-RAD T100 Thermal Cycler with conditions of an initial denaturation at 95 °C for 5 min, followed by 35 cycles at 95 °C for 30 s, annealing at 58/61/65 °C for 30 s and extension at 72 °C for 45 s, and then a final extension at 72 °C for 5 min. All the reads were assessed manually and genotypes of each site were identified by the Sanger sequencing peaks. Subsequently, we compared genotypes of each site identified by whole-genome resequencing and obtained by the Sanger sequencing for the same individuals.

### Supplementary Information

**Additional file 1.**

**Additional file 2.**

## Data Availability

Sequences were private from ENA with the Bioproject accession numbers PRJEB67694.

## Abbreviations

GZB: Guizhou black goat
YBG: Yunshang black goat
CG: Cashmere goat
IIG: Iran indigenous goat
TG: Tibetan goat
SCG: Shaanbei white cashmere goat
SKG: South Korean goat
WG: Wild goat
FG: French goat
MG: Morocco goat
SNP: Single nucleotide polymorphism
WGS: Whole-genome sequencing
θ_π_: Nucleotide diversity
*F*_ST_: Fixation index
XP-EHH: The Cross-Population Extended Haplotype Homozygousity
GO: Gene Ontology
KEGG: Kyoto Encyclopedia of Genes and Genomes
NJ: Neighbor-joining
PCA: Principle component analysis
LD: Linkage disequilibrium
