# Supplementary material for: Identification of genomic characteristics and selective signals in Guizhou black goat
Source: BMC Genomics. 2024 Feb 9;25:164. doi: 10.1186/s12864-023-09954-6 (PMC10854126; doi:10.1186/s12864-023-09954-6)
Supplement: Supplementary file 2 — Additional file 2. [file 12864_2023_9954_MOESM2_ESM.docx]

**Statement on confirming the Study in Accordance with ARRIVE guidelines**

1. **Study design**

Guizhou black goat (GZB) is one of the indigenous black goat breeds in the southwest region of Guizhou, China, which is an ordinary goat for mutton production. They are characterized by moderate body size, black coat, favorite meat quality with tender meat and lower odor, crude feed, and cold tolerance. In contrast, Iran indigenous goats, Yunshang black goats, and cashmere goats（Shaanbei white cashmere goats and Tibetan goats) show great differences from Guizhou black goats in reproduction, growth and development, disease resistance, and so on. Therefore, to find potential genomic evidence linking the domestication of Guizhou black goats with their breed characteristics, we performed large-scale sequencing and systematic comparisons between unrelated Iran indigenous goats (n=15) and Yunshang black goats (n=11), cashmere goats（Shaanbei white cashmere goats (n=9) and Tibetan goats (n=4)), respectively.

1. **Sample size**

We performed whole-genome resequencing on 30 unrelated individual GZB. Additionally, we downloaded 79 publicly available goat genome sequences from the European Bioinformatics Institute website (www.ebi.ac.uk/), including Iran indigenous goats (IIG, n=15), Morocco goats (MG, n=12), South Korean goats (SKG, n=12), Yunshang black goats (YBG, n=11), French goats (FG, n=10), Shaanbei white cashmere goats (SCG, n=9), wild goats (WG, n=6), and Tibetan goats (TG, n=4).

1. **Inclusion and exclusion criteria**
2. We exclude the individuals who are related.
3. We removed those INDELs in the VCF file using the following options: QUAL < 30.0 || QD < 2.0 || FS > 200.0 || SOR > 3.0 || MQRankSum < -12.5 || ReadPosRankSum < -20.0.
4. High-quality SNPs were identified according to the filtering criteria: QUAL < 30.0 || QD < 2.0 || FS > 60.0 || MQ < 40.0 || SOR > 3.0 || MQRankSum < -12.5 || ReadPosRankSum < -8.0.
5. Before detecting selection signals, we filtered the SNPs with minor allele frequency (MAF) < 0.05 and call rates < 0.90 and excluded sites with a missing rate > 20% using PLINK.
6. To avoid spurious selection signals, windows with less than 10 SNPs were discarded.
7. Putative selection targets were identified as the candidate regions in fully overlapping windows with high *F*_ST_ (*F*_ST_ > 99%) and θ_π_ (θ_π_ > 99%) values.
8. **Randomisation**

Randomized sampling was not used in this study. We collected 30 unrelated Guizhou black goats according to the pedigree records from the goat breed farms.

1. **Blinding**

The blind method was not used in this study.

1. **Outcome measures**
2. We first detected the quality of DNA.
3. We analyzed the sequencing quality and average sequencing depth for each sample.
4. We identified heritable variants (SNPs).
5. Neighbor-joining (NJ) trees, principal component (PCA), and ADMIXTURE and LD analysis were performed.

(5) We explored the genomic regions under selection in Guizhou black goats using two complementary methods: *F*_ST_ and θ_π_.

1. **Statistical methods**
2. Genomic DNA was sequenced using paired-end libraries on a DNBSEQ-T7 sequencer by BGI-Shenzhen, China.
3. The raw paired-end reads were filtered and trimmed using the FastQC with default parameters. High-quality reads were then aligned to the goat reference genome ARS1 using the Burrows-Wheeler Aligner (BWA) with the “bwa-mem” algorithms. SAMtools was used for sorting and indexing the aligned BAM files. Duplicates were removed by the MarkDuplicates module in GATK. Finally, we jointly used “HaplotypeCaller”, “CombineGVCFs”, and “GenotypeGVCFs” with default parameters in GATK to call variants, which generated genotype calls in Variant Call Format (VCF).
4. Principal component analysis (PCA) was carried out using the PLINK software (v1.07), and the first two eigenvectors were plotted in the ggplot2 package under the R platform. For phylogenetic tree analysis, PLINK was used to calculate the genetic distance matrix. Next, the phylogenetic tree was constructed with the neighbor-joining (NJ) algorithm in MEGA X and displayed with FigTree (v1.4.4). Moreover, the population ancestry was estimated using ADMIXTURE software (v1.3) with kinship (K) set from 2 to 9. The genome-wide linkage disequilibrium (LD) decay between pairwise SNPs was assessed and visualized using PopLDdecay software (v3.41).
5. We explored the genomic regions under selection in GZB using two complementary methods: *F*_ST_ and θ_π_. We used two methods including the fixation statistics (*F*_ST_) and population nucleotide diversity ratio θ_π_ to identify the selection signatures in GZB. The average *F*_ST_ and θπ were calculated by VCFtools (v0.1.13) in 100 kb windows sliding with a 10 kb step size between GZB and the other three populations. Putative selection targets were identified as the candidate regions in fully overlapping windows with high *F*_ST_ (*F*_ST_ > 99%) and θ_π_ (θ_π_ > 99%) values. The selected regions were annotated using Bedtools (v2.17.0). For each comparison, functional enrichments of the selected genes comprising the KEGG pathway and GO classes were analyzed using KOBAS-i to investigate the biological enrichment of genes under selective pressure. The terms with *p-values* smaller than 0.05 were considered statistically significant.
6. **Experimental animals**

All of the 30 GZB are reared in the same environment (Guizhou black goat breeding farms in Guizhou province, China). They are about one year old. These experimental samples were sow in good health.

1. **Experimental procedures**
2. Sample collection and sequencing

In this study, we obtained a total of 30 unrelated Guizhou black goats (GZB) based on pedigree records from the goat farms in Weining and Hezhang counties of Guizhou province. For each goat, the whole blood sample and ear tissue were collected for DNA extraction. The high-quality DNA was used for the whole-genome resequencing.

1. Identification of heritable variants
2. Population genetic structure and linkage disequilibrium

To assess the genetic structure among the pig breeds in this study, Neighbor-joining (NJ) trees, principal component analysis (PCA), and ADMIXTURE were performed.

1. Identifying selection signatures using *F*_ST_ and θ_π_ approaches

We used two methods including the fixation statistics (*F*_ST_) and population nucleotide diversity ratio θ_π_ to identify the selection signatures in GZB. Putative selection targets were identified as the candidate regions in fully overlapping windows with high *F*_ST_ (*F*_ST_ > 99%) and θ_π_ (θ_π_ > 99%) values.

1. GO terms and KEGG pathway enrichment analyses

For each comparison, we subsequently investigated the functions associated with the annotated genes undergoing positive selection by analyzing over-represented GO terms and KEGG pathway analysis.

1. **Results**

Here, we resequenced the whole genome of Guizhou black goat from 30 unrelated individuals breeding in the core farms. A total of 9,835,610 SNPs were detected, and 2,178,818 SNPs were identified specifically in this breed. The population structure analysis revealed that Guizhou black goat shared a common ancestry with Shaanbei white cashmere goat (0.146), Yunshang black goat (0.103), Iran indigenous goat (0.054), and Moroccan goat (0.002). However, Guizhou black goat showed relatively higher genetic diversity and a lower level of linkage disequilibrium than the other four goat breeds by the analysis of the nucleotide diversity, linkage disequilibrium decay, and runs of homozygosity. Based on FST and θπ values, we identified 645, 813, and 804 selected regions between Guizhou black goat and Yunshang black goat, Iran indigenous goat, and cashmere goat. There were 252, 290, and 324 genes in these selected regions, respectively. Functional annotation analysis revealed that these genes are potentially responsible for the immune response (e.g., *CD28, CD274, IL1A, TLR2,* and *SLC25A31*), humility-cold resistance (e.g., *HBEGF, SOSTDC1, ARNT,* and *EP300*), meat quality traits (e.g., *CHUK, GAB2,* and *EP300*), growth (e.g., *GAB2, DPYD,* and *CSF1*), fertility (e.g., *METTL15* and *MEI1*), and visual function (e.g., *PANK2* and *NMNAT2*) in Guizhou black goat.

1. **Objectives**

In this study, we identified many heritable variants and a suite of potential candidate genes that are involved in crucial biological processes such as growth, disease resistance, reproduction, and meat quality traits. These findings will facilitate the understanding of the germplasm characteristics and support further investigation of the mechanisms underlying selection in GZB.

1. **Ethical statement**

All animal procedures were approved by Guizhou University Subcommittee of Experimental Animal Ethics (EAE-GIU-2021-P009) and were conducted according to the rules of animal experimental ethics. The study is also reported in accordance with ARRIVE guidelines.

**13. Housing and husbandry**

All of the goats are reared in the same environment (Guizhou black goat breeding farms in Weining and Hezhang counties of Guizhou province). Light-dark cycle, room temperature, humidity, environmental purification, drinking water, and food supply were conducted by Guizhou black goat breeding farms.

**14. Animal care and monitoring**

All of the 30 unrelated Guizhou black goats used in this experiment were cared for and monitored by Guizhou black goat breeding farms in Weining and Hezhang counties of Guizhou province.

**15. Interpretation/scientific implications**

In the present study, we performed the whole-genome resequencing of 30 unrelated individual Guizhou black goats and conducted population structure, LD decay, and selection signatures analyses for the Guizhou black goat and three other populations (i.e., Iran indigenous goat, Yunshang black goat, and cashmere goat). We detected some heritable missense mutations and a suite of potential candidate genes that are involved in crucial biological processes such as growth, disease resistance, reproduction, and meat quality traits. These variations and genes related to phenotype and physiology are only analyzed based on genotype frequency. It is necessary to further determine the phenotype data and carry out association analysis. The variations and genes found in this study also need to be verified by corresponding biological function experiments. The variations and genes found in this study also need to be confirmed by further experimental verification of biological function.

**16. Data access**

Sequences are private from ENA with the Bioproject accession numbers PRJEB67694.

**17. Declaration of interests**

The authors declare no competing interests.
